# Commissioning and clinical implementation of Monaco treatment planning system on a compact pencil beam scanning proton therapy system

**DOI:** 10.1002/acm2.70587

**Published:** 2026-04-15

**Authors:** Xiangkun Xu, Xiaoda Cong, Jian Liang, An Qin, Shupeng Chen, Xiaoqiang Li, Weili Zheng, Peilin Liu, Raymond Dalfsen, James Dolan, Martin Soukup, Craig Stevens, Rohan Deraniyagala, Xuanfeng Ding

**Affiliations:** ^1^ Department of Radiation Oncology Corewell Health William Beaumont University Hospital Royal Oak Michigan USA; ^2^ Elekta AB Stockholm Sweden

**Keywords:** commissioning, proton therapy, Treatment planning system

## Abstract

**Background:**

Treatment planning system (TPS) requires comprehensive commissioning and validation prior to clinical use to ensure accurate dose calculation and safe clinical implementation.

**Purpose:**

To enable clinical use of the Monaco proton TPS, we commissioned it on a single‐room compact proton therapy system and implemented a clinically integrated workflow using daily synthetic CT (sCT) to track fractional dose delivery.

**Methods:**

The Monte Carlo (MC) dose calculation algorithm in Monaco TPS (version 6.1) was commissioned on an IBA Proteus ONE system. Commissioning included data acquisition, beam modeling, and model validation. Data included in‐air spot profiles for 70–227 MeV at five planes (+20 cm and +10 cm upstream, isocenter, −10 cm and −20 cm downstream), integral depth‐dose curves in water, and absolute monitor unit calibration. Model validation comprised: percent depth‐dose in water for monoenergetic beams using a 10 × 10 cm^2^ field across energies; output checks with standard cube plans mimicking diverse treatment sites and target volumes; and independent end‐to‐end testing with an IROC brain phantom. Clinically, a patient with basal cell carcinoma of the skin was treated with a two‐field IMPT plan delivering 60 Gy in 30 fractions. Probability‐based robust optimization required 100% target coverage under 3 mm setup and 3.5% range uncertainties. An in‐house sCT generated from daily CBCT images was used to evaluate dose delivery accuracy throughout treatment.

**Results:**

Distal range (R90, R80, R20) differences between Monaco and measurement were within 1 mm for 70–227.7 MeV. Output differences for all standard cube plans were within 3%. For the IROC brain phantom, the TLD‐to‐Monaco MC dose ratio was 1.00, with gamma passing rates of 94% (coronal) and 98% (sagittal). sCT‐based evaluation showed 99% CTV receiving ≥98% of the prescribed dose across the course.

**Conclusions:**

Monaco's proton MC beam model and dose calculation algorithm were commissioned and validated on IBA Proteus ONE for clinical use. The first patient (November, 2024) was successfully treated, with daily dose delivery accuracy monitored via an in‐house sCT platform.

## 1 INTRODUCTION

The treatment planning system (TPS) is a cornerstone of modern radiation oncology, serving as the central hub from patient simulation through treatment delivery. It provides a multidisciplinary platform for physicians, dosimetrists, physicists, and therapists to generate contours, optimize treatment plans, and transfer approved plans to the treatment machine. In proton therapy, the role of the TPS is particularly critical due to the unique physical characteristics of protons, notably the Bragg peak, which enables highly conformal dose delivery to tumors while sparing surrounding normal tissues.^1^ However, this precision introduces additional complexity, necessitating a robust and well‐validated TPS capable of accurate beam modeling, dose calculation, and treatment delivery.

Dose calculation algorithms play a central role in treatment accuracy. While pencil beam algorithms are computationally efficient, they may be less reliable in heterogeneous tissues such as lung^2^ or head‐and‐neck regions.^3^ Monte Carlo (MC) dose calculation, although computationally intensive, provide superior accuracy by explicitly simulating proton interactions.^4^ Several studies^3^, ^5^ have highlighted the clinical benefits of MC‐based dose calculation, particularly in anatomically complex sites. Therefore, in this study we focused on commissioning MC‐based dose calculation.

Beyond beam modeling and dose calculation, robust treatment planning is a key requirement in proton therapy. Static robust optimization strategies address setup and range uncertainties, but inter‐fractional robustness has become increasingly important in routine clinical practice. Proton beams are highly sensitive to anatomical changes, making daily dose tracking and validation essential. Synthetic CTs (sCT) generated from cone‐beam CT (CBCT) or other imaging modalities enable possibility of recalculation of the delivered dose and assessment of deviations from the planned distribution on the daily basis.^6^, ^7^ This approach supports adaptive therapy decisions by allowing clinicians to respond to changes such as tumor shrinkage or weight loss. Studies by Taasti et al.^8^ and Kaushik et al.^9^ have demonstrated the potential of sCT based dose validation, though most commercial TPS platforms do not yet offer this functionality as a standard clinical feature, necessitating in‐house development.

Given the central role of TPS in proton therapy, it is essential to thoroughly document commissioning and validation procedures when a new platform becomes commercially available. Such validation typically includes dose calculation accuracy, Bragg peak and independent end‐to‐end testing, such as IROC phantom irradiations. Standardized reporting of these processes facilitates best‐practice sharing and supports the safe integration of new TPS modules into routine clinical workflows.^10^, ^11^

In this study, we report the first commissioning and clinical implementation of the Monaco proton therapy module (version 6.1, FDA‐cleared in 2021) on a compact proton therapy system, IBA Proteus ONE. Furthermore, the clinical treatment was monitored using daily dose tracking using an in‐house developed sCT platform, enabling safe and precision proton treatment throughout the treatment course.

## 2 METHODS

### 2.1 Proton therapy system

All measurements and clinical treatments were performed on a compact single‐room proton therapy system (Proteus ONE, IBA, Louvain‐la‐Neuve, Belgium). The system employs a superconducting cyclotron with a maximum proton energy of 227.7 MeV and a pencil beam scanning (PBS) nozzle designed for dynamic spot delivery. The gantry provides 220° of rotation and is integrated with a six‐degree‐of‐freedom robotic couch. The system supports a maximum field size of 20 × 24 cm^2^ at isocenter, with layer switching time of <1 s.

### 2.2 Monaco proton treatment planning system

The Monaco treatment planning system (TPS, version 6.1, Elekta AB, Stockholm, Sweden), was commissioned for proton PBS delivery. The TPS includes both a pencil beam (PB) algorithm and a MC dose calculation engine; In this study, we decided to focus on the MC dose calculation commission. The beam commissioning data, including in‐air beam spot profile, integrated depth dose (IDD) curves and MU calibration data, collected from the Proteus ONE to generate a physics beam model in Monaco TPS. A CT‐to‐electron density table was created, and a look up table in Monaco TPS is used to map the electron density to tissue types or other materials. The stopping power of the tissue/material from ICRU 49 will be used in the TPS for proton dose calculation. The mass density not included in the list will be treated as a mass‐fraction weighted mixture of the two material with closest mass density. Probability‐based robust optimization was integrated into the Monaco TPS, with user‐defined parameters such as setup and range uncertainties, as well as the percentage of probability to meet such criteria.

### 2.3 Measurement of beam commissioning data

#### 2.3.1 In‐air beam spot profiles

Lateral spot profiles were measured at five longitudinal positions relative to isocenter (−20, −10, 0, +10, and +20 cm) in air with Gantry at 0° for proton proton energies in 5 MeV increments ranging from 70 to 227.7 MeV. 0 is at isocenter, +20 and +10 cm is the upstream relative to isocenter, −20 and −10 cm is the downstream relative to isocenter. Measurements were performed using a scintillation‐based 2D detector (Lynx PT, IBA Dosimetry GmbH, Schwarzenbruck, Germany), which provides 0.5 mm high spatial resolution beamlet images suitable for Gaussian fitting of lateral distributions. The calibration of Lynx^12^ includes: (1) uniformity correction using a Co60 beam to ensure the maximum error within ± 2% from the water tank reference and (2) camera geometry calibration was performed using a metal grid plate, which was performed by the manufacturer before spot profile measurement.

#### 2.3.2 Integrated depth dose curve

IDD curves were measured at gantry 0° with a large parallel plate chamber (12 cm in diameter, Stingray, IBA Dosimetry GmbH, Schwarzenbruck, Germany) mounted in a motorized water phantom (Blue Phantom^2^, IBA Dosimetry GmbH, Schwarzenbruck, Germany). The IBA Ion Chamber was used as the reference chamber to synchronize the signal and data collection. When acquiring IDD curves, variable scanning speed was applied with 3–5 mm/s in the flat region and 0.2 mm/s in the Bragg peak region of the curve. Single spot and various energy layers from 70 to 227.7 MeV with 5 MeV increments were applied in the IBA ProteusONE physics mode. The dose rate is set between 0.5 and 2 nA, depending on the energy.

#### 2.3.3 Absolute dose calibration

The absolute dose per monitor unit (MU) was calibrated on measurements in a uniform monoenergetic single‐layer field. For each energy, a uniform 10 × 10 cm^2^ field was generated with spots arranged in a uniform grid with 2.5 mm center‐to‐center spacing. Each spot was assigned to 1 MU, and dose was measured at 2.5–7 cm depth depending on the energy layer in a water tank with a parallel‐plate ionization chamber (PPC05, IBA Dosimetry GmbH, Schwarzenbruck, Germany) calibrated by an Accredited Dosimetry Calibration Laboratory (ADCL). In IBA proton system, the MU is defined as 300 counts per MU from the reading of primary ion chamber. Therefore, the absolute dose to water at the reference depth was linked to the MU. In TPS dose calculation, a relative biological effectiveness factor of 1.1 was applied.

### 2.4 Proton beam modeling

As the beam modeling was performed by Elekta staff, prior to full beam model commissioning in the Monaco TPS, a series of independent sanity checks were performed to verify the internal consistency of the measured data and modeling results. The checks focused on two key dosimetric parameters: the spot profile in air and the IDD in water.

#### 2.4.1 Spot profile verification

The Gaussian sigma width in both the X and Y directions were extracted separately by fitting each spot profile to a Single‐Gaussian model. The lateral spot size with proton energy at isocenter was examined to confirm beam optics consistency and verify the expected trend of decreasing spot size with increasing energy. Any deviation greater than 0.2 mm between measurement and beam modeling was flagged for review.

#### 2.4.2 Integrated depth dose verification

Each IDD was normalized to 100% at maximum dose. The measured range of 90%, 80% and 20% (R_100_, R_90_, R_80_, and R_20_) on the distal edge of the Bragg peak were compared to TPS‐calculated data to confirm the accuracy of beam modeling.

### 2.5 TPS model validation

The accuracy of the Monaco physics beam model and the dose calculation algorithm was evaluated through a series of independent validation tests, including range verification, output consistency checks, and end‐to‐end dosimetric testing. These steps were designed to confirm that the TPS reliably reproduces measured beam data and performs accurately in clinically representative conditions.

#### 2.5.1 Range validation

Range accuracy was evaluated using pristine Bragg peaks delivered with a 10 × 10 cm^2^ uniform spot grid, with 2.5 mm spacing between spots and 1 MU per spot. This field was measured using the Zebra multilayer ionization chamber (MLIC, IBA Dosimetry GmbH, Schwarzenbrück, Germany), which consists of multiple ion chambers stacked along the beam axis with 2 mm pitch. The Zebra system provides high‐resolution percentage depth‐dose (PDD) measurements across the entire Bragg curve. For proton energies from 70 to 227.7 MeV, the TPS‐calculated PDDs were normalized to the maximum point of the measured Bragg peak, and the distal 90%, 80%, and 20% dose range (R90, R80, R20) was extracted for comparison. An agreement within ± 1 mm was required across the entire energy spectrum^11^.

#### 2.5.2 Output validation

Absolute dose accuracy was tested using standard cube phantom plans designed to mimic clinical target sizes, depths, and modulation widths. Each plan delivered a uniform dose to a water phantom, with chamber measurements acquired using a calibrated parallel‐plate ionization chamber (PPC05, IBA Dosimetry GmbH, Schwarzenbrück, Germany) at the isocenter. According to International Atomic Energy Agency technical reports serious No.398, after considering calibration uncertainty, dosimeter reading, influence quantity correction, beam quality correction, and other relevant factors, the combined standard uncertainty for determining dose to water with the above chamber is at 1.7%. For non–range‐shifter configurations, measurements were performed at reference depths between 5 and 21 cm depending on the cube plan. In our proton machine, a 3.5 cm thick Lexan range shifter was used, and the range shifter can be moved along with the snout with positioning spanning between 17.30 and 45.10 cm. For range‐shifter validation, the R14M12F5 cube plan was delivered, which represents a 14 cm range, 12 cm modulation width, and 5 cm field size. Absolute dose was measured at 1 cm depth in water downstream of the range shifter, a geometry that is particularly sensitive to multiple Coulomb scattering and secondary particle contributions. The dose at the machine isocenter, which was set at 7 cm depth of water downstream of the range shifter, was also measured.

#### 2.5.3 End‐to‐end testing

The integrity and accuracy of the entire clinical treatment workflow from CT simulation, contour, planning, transfer to Oncology Information System, to final treatment delivery were validated through an independent end‐to‐end test using the Imaging and Radiation Oncology Core (IROC) head phantom. The phantom includes tissue‐equivalent materials and dosimetry inserts accommodating thermoluminescent dosimeters (TLDs) and radiochromic film in predefined target and organ‐at‐risk (OAR) locations. A clinical plan was generated in Monaco TPS, exported to the Proteus ONE system, and delivered under routine treatment conditions. Following irradiation, TLDs were analyzed and compared against TPS MC dose calculation at the corresponding locations, with agreement within ± 5% considered acceptable. Film dosimetry in coronal and sagittal planes was analyzed using gamma index criteria (5%/3 mm). Gamma pass rates of ≥85% were required for successful validation.^13^ This comprehensive test verified the accuracy of imaging, TPS dose calculation, plan export, and delivery within a clinically realistic scenario.

Besides IROC phantom testing, we also performed clinically relevant plans including head and neck, thymus, and prostate, and patient specific QA was implemented to confirm the TPS planning accuracy. The QA measurements were conducted using a 2D ion chamber array (MatriXXONE, IBA Dosimetry GmbH, Schwarzenbrück, Germany). The detector plan was at 0.7 cm depth. The measured planar dose distribution was compared with Monaco MC‐calculated doses using a relative gamma index analysis with 3%/3 mm criteria.

The above validation procedures confirmed that the Monaco proton physics beam model accurately reproduces measured beam characteristics, delivers consistent output across a range of clinical conditions, and performs reliably in an end‐to‐end workflow.

### 2.6 Clinical implementation of Monaco proton TPS

A patient with basal cell carcinoma of the skin was selected for the first clinical treatment. The prescribed dose was 60 Gy in 30 fractions. An IMPT plan was generated in Monaco with two fields (left anterior oblique and left superior posterior oblique). The 3.5 cm thick Lexan range shifter with lowest snout position at 17.3 cm was applied. The plan was optimized with probability‐based robust optimization with parameters at 95% of prescribed 60 Gy to cover 100% clinical target volume (CTV) in the presence of 3 mm setup and 3.5% range uncertainties. Patient‐specific quality assurance (QA) was performed to verify the accuracy of dose calculation and plan delivery before clinical treatment. The QA measurements were conducted with the same method described in Section 2.5.3. Daily dose verification was performed using an in‐house sCT platform, which employed deformable registration and machine learning algorithms to generate sCT images from daily CBCT.^14^ Briefly, the sCT model was developed using paired CT/CBCT data from 35 patients treated at our institution. To reduce anatomical mismatch between image pairs, deformable registration was performed prior to training. The model was based on a GAN framework with Res‐UNet‐related improvements, and data augmentation included random 3D B‐spline transformations during pre‐training as well as on‐the‐fly 2D similarity and B‐spline deformations during training. The sCT platform was validated by comparing the dose agreement with simulation CT. As the These sCTs were imported into Monaco for dose recalculation to assess daily target coverage and organ‐at‐risk sparing.

## 3 RESULTS

### 3.1 Verification of beam modeling

**FIGURE 1:**
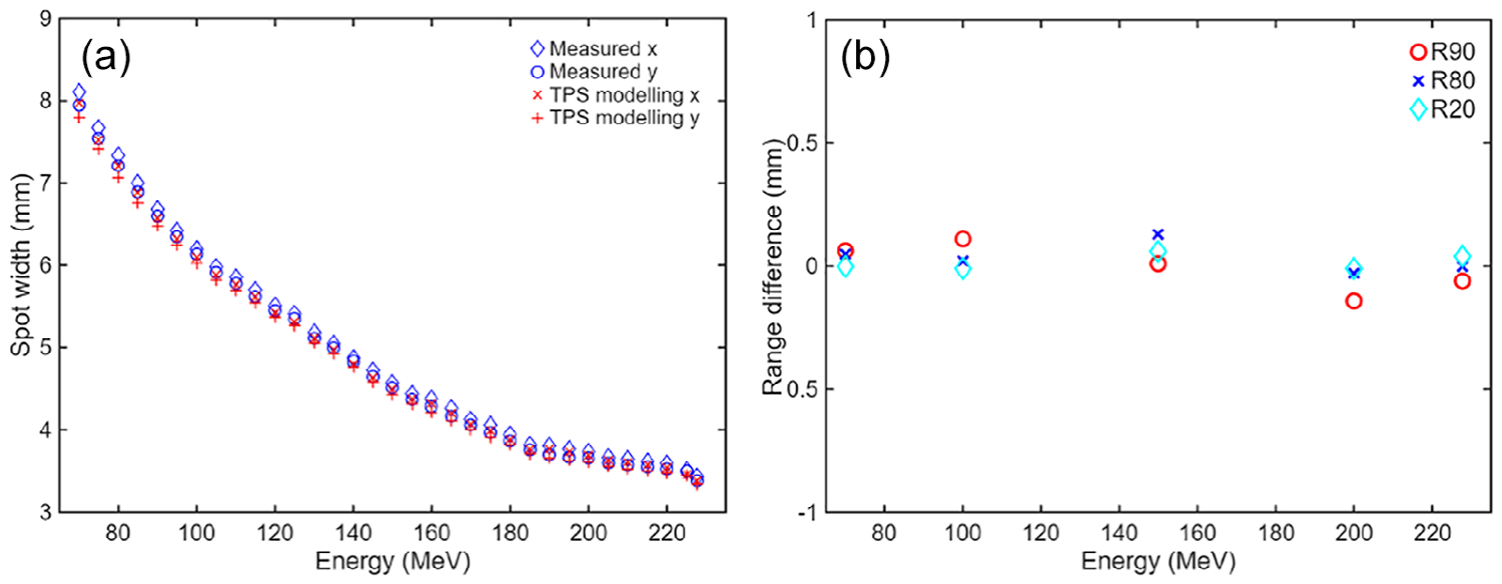
(a) Spot profile width comparison between measurement and TPS beam modeling at proton treatment machine isocenter. (b) Range comparison between IDD measurement and TPS modeling for selective energy at 70, 100, 150, 200, and 227.7 MeV.

Figure 1a shows the deviation of Gaussian sigma width of beam spot profile at the isocenter along x and y directions between the measurement and TPS beam modeling from 70 to 227.7 MeV. The spot width is decreasing from 8 to 3.5 mm with increasing energy. The deviation among all the energies is within 0.15 mm. Figure 1b shows the range difference of R_90_, R_80_, and R_20_ between IDD measurement and TPS beam modeling data for selective energies from 70 to 227.7 MeV. The range difference is defined as the TPS modeling range minus measurement range from water. All range difference is within 0.25 mm, confirming the beam modeling accuracy.

### 3.2 TPS validation

**FIGURE 2:**
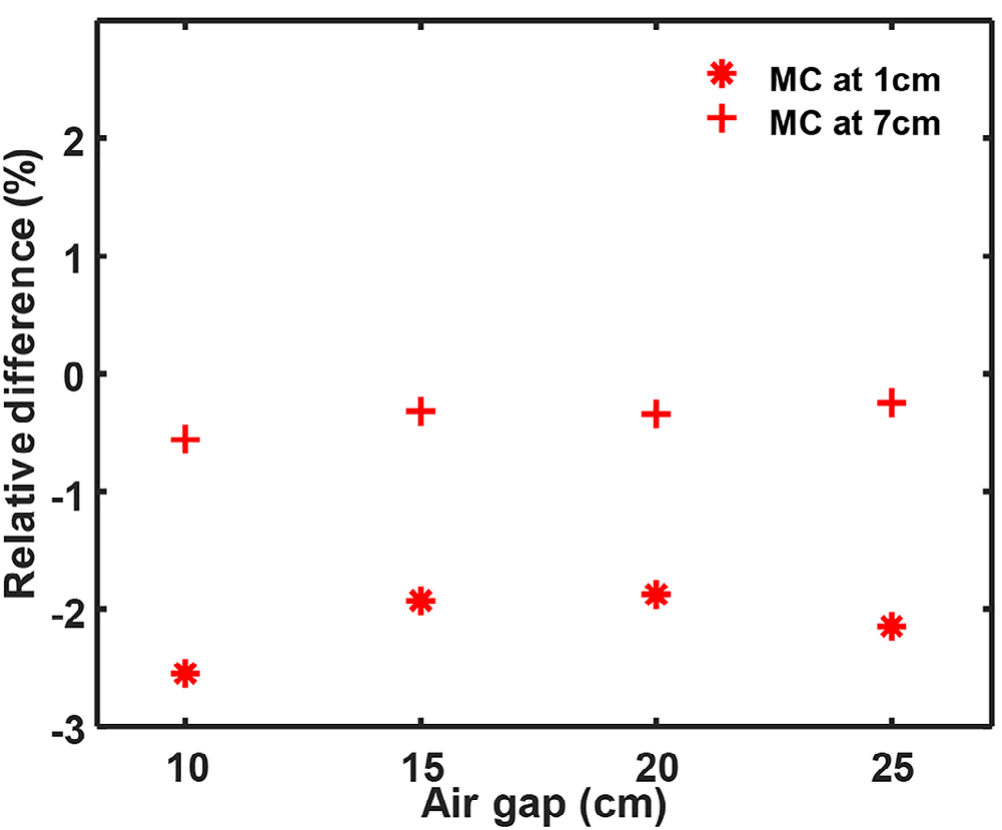
Output validation for Monte Carlo (MC) dose calculation algorithms at different air gaps when range shifter was applied. The measurement data was acquired at depths of 1 and 7 cm relative to water tank surface for standard cube plan R14M12F5 with range shifter, and the machine isocenter was set at depth of 7 cm.

1. We compared R90, R80 and R20 between Zebra measurement and calculation with MC in Monaco TPS for selected energies shown in Table 1. The difference is defined as the TPS calculation with 1 mm dose grid minus Zebra measurement. The deviations among all the selected energies are less than 1 mm, which is aligned with AAPM TG recommendation.^11^
2. The output validation demonstrated good agreement between measured and Monaco‐calculated doses across all test configurations. For standard cube phantom plans without range shifter (Table 2), the differences between measured and TPS calculated absolute doses were within ± 2% across the full range of proton energies and field sizes. The results confirmed consistent beam output and linear MU response throughout the clinical energy spectrum. For the rang‐shifter conditions, the R14M12F5 cube plan was used to represent a clinically relevant shallow treatment geometry. The measured dose at depths of 1 and 7 cm agreed with the TPS‐calculated dose within ± 2.6% and ± 1% at air gaps from 10 to 25 cm, as shown in Figure 2, respectively, demonstrating that Monaco TPS accurately modeled the additional scattering and energy degradation introduced by the range shifter.
3. For IROC brain phantom, the ratio of the TLD measured dose to the Monaco MC calculated dose is 1 at both posterior and anterior locations. The gamma index on coronal and sagittal film planes is 94% and 98% when using dose/distance agreement criteria of 5% and 3 mm, respectively. The phantom irradiation results meet the criteria of IROC.
4. We have examined three patients with six treatment beams for patient specific QA for both with and without range shifters. Our patient specific QA results showed the gamma index passing rate at 99.73 ± 0.61% and the point dose difference between planning and measurement is at 1.4 ± 1.3% (*n* = 6), highlighting the Monaco TPS calculation accuracy.

**TABLE 1:** Comparison between Zebra measured and Monte Carlo calculated R90, R80, and R20 (mm) for selected beam energy.

**Energy (MeV)**	**Difference R90**	**Difference R80**	**Difference R20**
70	0.2	0.1	−0.1
100	0	0.5	0
150	0.5	0.3	0.5
200	−0.1	0.1	0.9
227.7	−0.2	−0.1	0.8

**TABLE 2:** Output validation for Monte Carlo (MC) algorithms.

**Cube phantoms**	**Iso depth (cm)**	**Measure depth (cm)**	**Measure (cGy)**	**MC (cGy)**	**MC vs. measure**
R26M10F10	21	21	201.79	198.2	1.8%
R23M6F18	20	20	202.37	199.3	1.5%
R20M10F10	15	15	202.59	198.9	1.9%
R9.5M3F4	8	8	199.11	201.5	−1.2%
R7M4F8	5	5	201.36	199.9	0.7%

### 3.3 Clinical implementation

**FIGURE 3:**
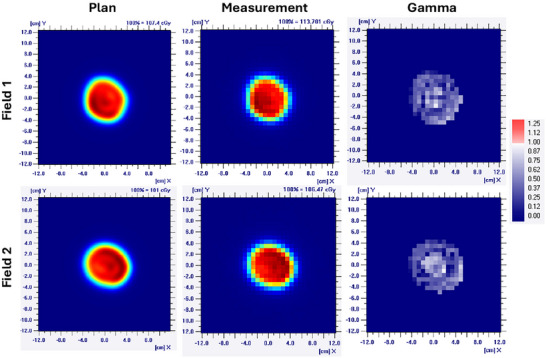
Patient specific QA results. The 2D dose distribution from treatment plan was compared to measurement data from 2D ionization chamber array through gamma analysis with a 3% dose difference and 3 mm distance to agreement.

For the basal cell carcinoma patient plan, QA measurements, shown in Figure 3, confirmed that both fields achieved gamma pass rates at 100% and a point dose agreement within 2%. These results validated the MC dose calculation accuracy for shallow targets.

**FIGURE 4:**
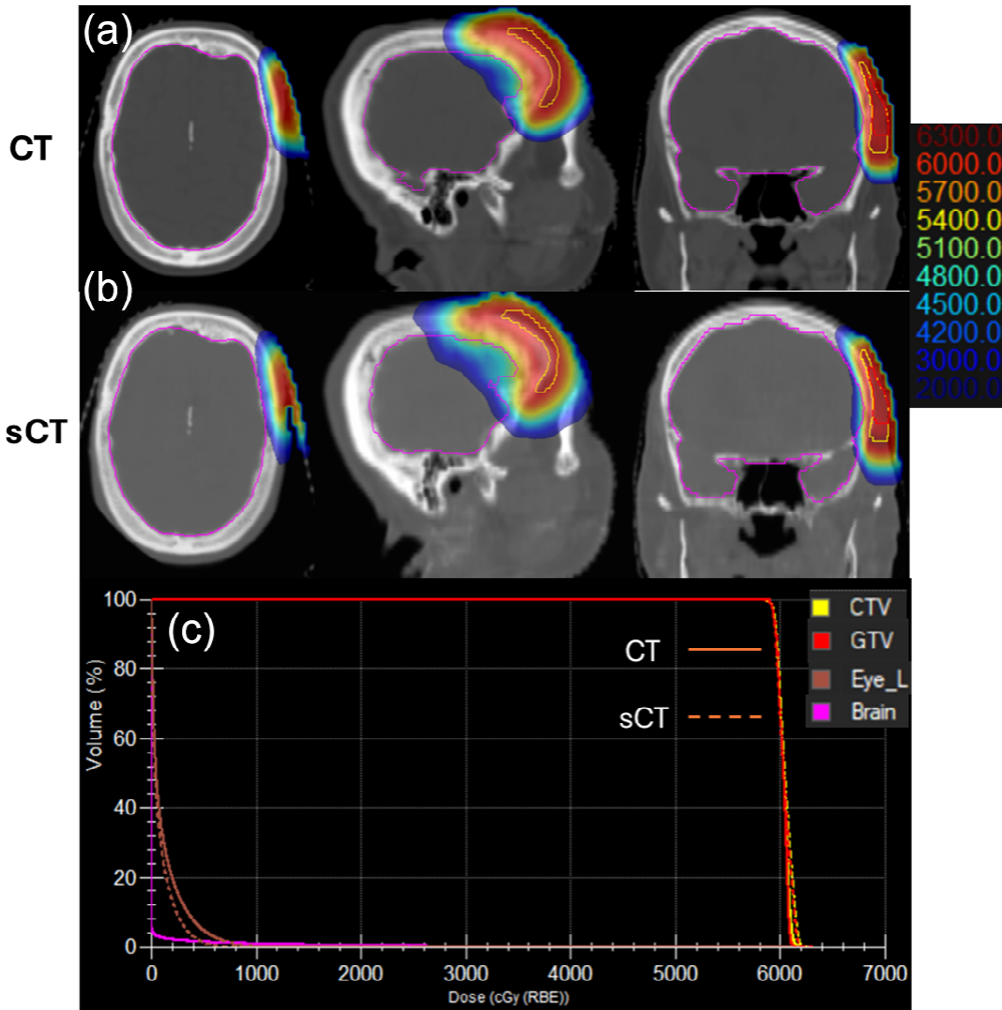
Dose comparison between planning CT and synthetic CT (sCT). (a, b) representative slices of dose distribution based on planning CT and synthetic CT for the basal cell carcinoma patient with MC algorithm, respectively. (c) The dose volume histogram comparison for targets and organs at risk.

Figure 4a,b present the dose distribution calculated on the planning CT for the initial planning and sCT for the fifth fraction, shown in axial, sagittal, and coronal planes, respectively. The results demonstrated visually indistinguishable agreement between the two calculations, with only minor localized differences near high‐gradient regions at the skin‐air surface, due to the skin contouring variation caused by mask positioning. No systematic dose shift or geometric distortion was observed in the sCT‐based dose calculation, confirming the geometric fidelity of the deformable registration and machine‐learning synthesis process. DVHs for the planning CT‐ and sCT‐ dose distribution were compared for the CTV, GTV and relevant organs at risk, left eye and brain, as shown in Figure 3c. The CTV coverage was maintained with D98 = 5934 cGy (CT) versus 5918 cGy (sCT) and Dmean = 6033 cGy versus 6046 cGy, respectively. Differences in OAR doses were negligible. These results confirm that the sCT‐based recalculations accurately reproduce the planned dose distribution and maintain clinically equivalent target coverage and OAR sparing. The consistent agreement in both spatial dose maps and DVH metrics supports the feasibility of using the sCT platform for daily dose tracking and validation in the routine proton therapy workflow.

**FIGURE 5:**
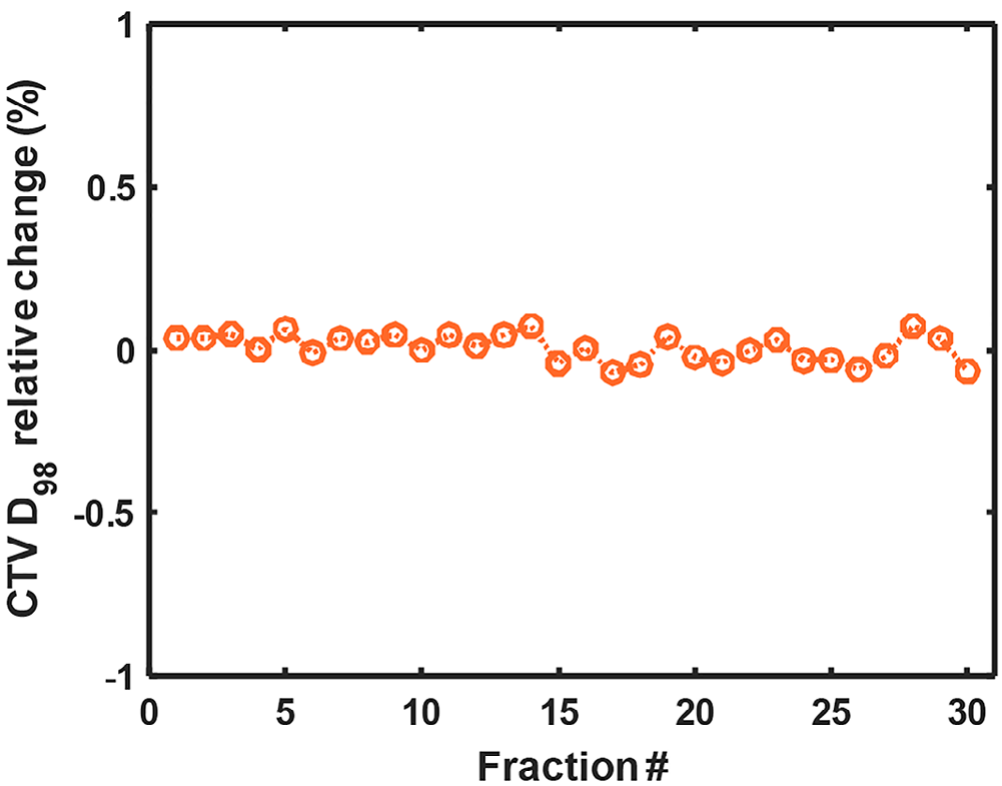
Tracking of D_98_ in CTV relative to the planning dose with daily synthetic CT image.

Following the sCT workflow, we tracked the dose at 98% coverage of CTV (D98) for 30 fractions (Figure 5). The D98 variation is within 0.2% among all fractions, suggesting robust dose delivery across the treatment course.

## 4 DISCUSSION

This study presents the first commissioning, validation, and clinical implementation of the Monaco proton therapy module on a compact Proteus ONE PBS system. The results demonstrated that the physics beam model and MC dose calculation engine achieved agreement within clinical tolerance across all commissioning tests, including range, output, and end‐to‐end validations. The distal range of pristine Bragg peaks agreed within 1 mm between Monaco and measurements, and the absolute dose difference was within 3% for all cube plans, including those delivered with a range shifter. The successful completion of IROC end‐to‐end testing and the first successful patient treatment confirmed the accuracy and robustness of the entire planning and delivery chain. Furthermore, integration of the in‐house sCT platform with Monaco enabled daily dose tracking, ensuring consistent target coverage throughout the treatment course.

Modern TPS, such as Monaco, now incorporate optimization algorithms capable of handling complex clinical scenarios through robust optimization and biological or multi‐criteria approaches. Robust optimization, as implemented in this work, explicitly accounts for setup and range uncertainties during the planning process, ensuring adequate CTV coverage even under worst‐case perturbations. Additional techniques provided by TPS such as layer repainting, volumetric rescanning, and LET‐based biological optimization could further enhance plan quality and robustness. These advanced techniques rely heavily on accurate dose computation and fast optimization algorithms, underscoring the importance of a reliable and validated TPS framework for clinical deployment. The field of radiation oncology is continually evolving, with new treatment technologies such as FLASH radiotherapy,^15^ spot‐scanning proton arc therapy (SPArc),^16^, ^17^ and spatial fractionation radiotherapy^18^ pushing the boundaries of what is possible in cancer treatment. The development of TPS for these emerging technologies involves not only adapting existing algorithms but also creating new ones to address the unique challenges they present.

One of the most significant findings of this work is the successful implementation of daily dose validation using sCT images derived from CBCT. Proton therapy is inherently sensitive to anatomical and setup variations due to the finite range and sharp distal fall‐off of the Bragg peak. Without daily verification, even small anatomical changes can cause clinically relevant dose discrepancies. By recalculating daily dose distributions on sCTs, clinicians can monitor dose consistency and detect deviations in target coverage or organ‐at‐risk (OAR) sparing. This capability provides a foundation for future adaptive proton therapy, where treatment plans can be modified in response to systematic anatomical changes such as tumor shrinkage or patient weight loss. The demonstrated agreement between planning CT and sCT dose distributions supports the feasibility of this approach and highlights the role of robust optimization and image‐based dose tracking in maintaining treatment fidelity over the course of therapy. Considering the scope of current TPS commissioning report, the fully detailed technical description and validation of the sCT platform is limited.

While this study successfully established and validated the Monaco proton TPS on a compact PBS system, several areas warrant further investigation. First, additional IROC end‐to‐end tests using different phantoms (e.g., lung, liver, and head‐and‐neck) would provide a more comprehensive verification of TPS performance across varied anatomical geometries and heterogeneities. Second, the accuracy of image registration, particularly deformable image registration (DIR) used in the sCT generation, should be quantitatively assessed, as registration uncertainties can propagate into the daily dose calculation. Incorporating deformable dose accumulation and evaluating its dosimetric impact will be essential for clinical adaptive workflows. Furthermore, future work will explore larger patient cohorts to assess plan robustness and sCT‐based dose validation under different clinical scenarios.

## 5 CONCLUSIONS

The commissioned Monaco proton TPS was within clinical tolerances. The successful clinical implementation of Monaco on a compact PBS system demonstrated accurate beam modeling and robust dose calculation. The integration of sCT–based dose tracking represents an important step toward adaptive proton therapy, enabling continuous monitoring of delivered dose and maintaining treatment precision throughout the course. Continued validation of image registration accuracy, adaptive optimization techniques, and multi‐site phantom testing will further strengthen the clinical reliability of this system.

## AUTHOR CONTRIBUTIONS

The authors confirm contribution to the article as follows. Study conception and design: X. Xu, C. Stevens, and X. Ding; Data collection: X. Xu, X. Cong, J. Liang, A. Qin, S. Chen, X. Li, W. Zheng, P. Liu, and X. Ding; Analysis and interpretation of results: X. Xu, X. Cong, J. Liang, A. Qin, S. Chen, X. Li, W. Zheng, P. Liu, R. Dalfsen, J. Dolan, M. Soukup, C. Steven, R. Deraniyagala, and X. Ding; Draft manuscript preparation: X. Xu and X. Ding. All authors reviewed the results and approved the final version of the manuscript.

## CONFLICT OF INTEREST STATEMENT

Xuanfeng Ding received honoraria from IBA and the Elekta Speaker Bureau outside the scope of the work presented here. Xuanfeng Ding and Xiaoqiang Li are inventors on a patent related to Particle Arc Therapy, which is assigned to Corewell Health. A related license agreement exists with IBA.

## ETHICS STATEMENT

The study is approved by IRB 2017–455.

## Data Availability

The data used in this study are restricted to internal research purposes and are not publicly available. Requests for data access may be directed to the corresponding author.

## References

[1] Newhauser WD , Zhang R . The physics of proton therapy. Phys Med Biol. 2015;60(8):R155. doi:10.1088/0031‐9155/60/8/R155 25803097 10.1088/0031-9155/60/8/R155 PMC4407514

[2] Taylor PA , Kry SF , Followill DS . Pencil beam algorithms are unsuitable for proton dose calculations in lung. Int J Radiat Oncol Biol Phys. 2017;99(3):750‐756. doi:10.1016/j.ijrobp.2017.06.003 28843371 10.1016/j.ijrobp.2017.06.003 PMC5729062

[3] Sasidharan BK , Aljabab S , Saini J , et al. Clinical Monte Carlo versus pencil beam treatment planning in nasopharyngeal patients receiving IMPT. Int J Part Ther. 2019;5(4):32‐40. doi:10.14338/IJPT‐18‐00039.1 31773039 10.14338/IJPT-18-00039.1 PMC6871622

[4] Paganetti H , Jiang H , Parodi K , Slopsema R , Engelsman M . Clinical implementation of full Monte Carlo dose calculation in proton beam therapy. Phys Med Biol. 2008;53(17):4825. doi:10.1088/0031‐9155/53/17/023 18701772 10.1088/0031-9155/53/17/023

[5] Maes D , Bowen SR , Fung A , et al. Dose comparison between proton pencil beam and Monte Carlo dose calculation algorithm in lung cancer patients. Int J Radiat Oncol Biol Phys. 2017;99(2):E694. doi:10.1016/j.ijrobp.2017.06.2275

[6] Thummerer A , Zaffino P , Meijers A , et al. Comparison of CBCT based synthetic CT methods suitable for proton dose calculations in adaptive proton therapy. Phys Med Biol. 2020;65(9). doi:10.1088/1361‐6560/ab7d54 10.1088/1361-6560/ab7d54 32143207

[7] Kazemifar S , Barragán Montero AM , Souris K , et al. Dosimetric evaluation of synthetic CT generated with GANs for MRI‐only proton therapy treatment planning of brain tumors. J Appl Clin Med Phys. 2020;21(5):76‐86. doi:10.1002/acm2.12856 32216098 10.1002/acm2.12856 PMC7286008

[8] Taasti VT , Hattu D , Peeters S , et al. Clinical evaluation of synthetic computed tomography methods in adaptive proton therapy of lung cancer patients. Phys Imaging Radiat Oncol. 2023;27:100459. doi:10.1016/j.phro.2023.100459 37397874 10.1016/j.phro.2023.100459 PMC10314284

[9] Kaushik S , Vatterodt N , Ödén J , Fredriksson A , Korreman SS . Toma‐Dasu I. Synthetic computed tomography techniques for adaptive proton therapy in head and neck cancers. Phys Imaging Radiat Oncol. 2025;36:100847. doi:10.1016/j.phro.2025.100847 41141228 10.1016/j.phro.2025.100847 PMC12550569

[10] Farr JB , Moyers MF , Allgower CE , et al. Clinical commissioning of intensity‐modulated proton therapy systems: report of AAPM Task Group 185. Med Phys. 2021;48(1):e1‐e30. doi:10.1002/mp.14546 33078858 10.1002/mp.14546

[11] Arjomandy B , Taylor P , Ainsley C , et al. AAPM task group 224: comprehensive proton therapy machine quality assurance. Med Phys. 2019;46(8):e678‐e705. doi:10.1002/mp.13622 31125441 10.1002/mp.13622

[12] Russo S , Mirandola A , Molinelli S , et al. Characterization of a commercial scintillation detector for 2‐D dosimetry in scanned proton and carbon ion beams. Phys Med. 2017;34:48‐54. doi:10.1016/j.ejmp.2017.01.011 28118950 10.1016/j.ejmp.2017.01.011

[13] Taylor PA , Kry SF , Alvarez P , et al. Results from the imaging and radiation oncology core Houston's anthropomorphic phantoms used for proton therapy clinical trial credentialing. Int J Radiat Oncol. 2016;95(1):242‐248. doi:10.1016/j.ijrobp.2016.01.061 10.1016/j.ijrobp.2016.01.061 PMC4834872 27084644

[14] Qin A , Xu J , O'Connell N , Ding X , Chen S , Thill D . The clinical usability, efficiency, and accuracy of combining deep‐learning‐based synthetic CT and auto‐contouring for daily CBCT‐based online adaptive radiation therapy. Med Phys. 2023;50(6):e210. doi:10.1002/mp.16525

[15] Vozenin MC , Bourhis J , Durante M . Towards clinical translation of FLASH radiotherapy. Nat Rev Clin Oncol. 2022;19(12):791‐803. doi:10.1038/s41571‐022‐00697‐z 36303024 10.1038/s41571-022-00697-z

[16] Liu P , Cong X , Liang J , et al. First clinical implementation of step‐and‐shoot proton arc therapy for head and neck cancer treatment. Int J Part Ther. 2025;16:100749. doi:10.1016/j.ijpt.2025.100749 40474940 10.1016/j.ijpt.2025.100749 PMC12138570

[17] Ding X , Li X , Zhang JM , Kabolizadeh P , Stevens C , Yan D . Spot‐scanning proton arc (SPArc) therapy: the first robust and delivery‐efficient spot‐scanning proton arc therapy. Int J Radiat Oncol Biol Phys. 2016;96(5):1107‐1116. doi:10.1016/j.ijrobp.2016.08.049 27869083 10.1016/j.ijrobp.2016.08.049

[18] Yan W , Khan MK , Wu X , et al. Spatially fractionated radiation therapy: history, present and the future. Clin Transl Radiat Oncol. 2020;20:30‐38. doi:10.1016/j.ctro.2019.10.004 31768424 10.1016/j.ctro.2019.10.004 PMC6872856

